# Do synthesis centers synthesize? A semantic analysis of topical diversity in research

**DOI:** 10.1016/j.respol.2020.104069

**Published:** 2021-01

**Authors:** Edward J. Hackett, Erin Leahey, John N. Parker, Ismael Rafols, Stephanie E. Hampton, Ugo Corte, Diego Chavarro, John M. Drake, Bart Penders, Laura Sheble, Niki Vermeulen, Todd J. Vision

**Affiliations:** a School of Human Evolution and Social Change, Arizona State University and Vice Provost for Research and Professor, Heller School for Social Policy and Management, Brandeis University; b School of Sociology, University of Arizona; c Department of Sociology and Geography, University of Oslo; d Centre for Science and Technology Studies, Leiden University; e Center for Environmental Research, Education and Outreach, Washington State University; f Department of Media and Social Sciences, University of Stavanger; g private consultancy; h Odum School of Ecology and Center for the Study of Infectious Diseases, University of Georgia; i Department of Health, Ethics, and Society, Care and Public Health Research Institute (CAPHRI), Maastricht University; j School of Information Sciences, Wayne State University, Duke Network Analysis Center, Social Science Research Institute (SSRI), Duke University; k Science, Technology, and Innovation Studies, University of Edinburgh; l Department of Biology and School of Information and Library Sciences, University of North Carolina at Chapel Hill

**Keywords:** Synthesis, Diversity, Innovation, Interdisciplinary Research, Creativity, Semantic Analysis, Scientific Collaboration

## Abstract

Synthesis centers are a form of scientific organization that catalyzes and supports research that integrates diverse theories, methods and data across spatial or temporal scales to increase the generality, parsimony, applicability, or empirical soundness of scientific explanations. Synthesis working groups are a distinctive form of scientific collaboration that produce consequential, high-impact publications. But no one has asked if synthesis working groups synthesize: are their publications substantially more diverse than others, and if so, in what ways and with what effect? We investigate these questions by using Latent Dirichlet Analysis to compare the topical diversity of papers published by synthesis center collaborations with that of papers in a reference corpus. Topical diversity was operationalized and measured in several ways, both to reflect aggregate diversity and to emphasize particular aspects of diversity (such as variety, evenness, and balance). Synthesis center publications have greater topical variety and evenness, but less disparity, than do papers in the reference corpus. The influence of synthesis center origins on aspects of diversity is only partly mediated by the size and heterogeneity of collaborations: when taking into account the numbers of authors, distinct institutions, and references, synthesis center origins retain a significant direct effect on diversity measures. Controlling for the size and heterogeneity of collaborative groups, synthesis center origins and diversity measures significantly influence the visibility of publications, as indicated by citation measures. We conclude by suggesting social processes within collaborations that might account for the observed effects, by inviting further exploration of what this novel textual analysis approach might reveal about interdisciplinary research, and by offering some practical implications of our results.

## Highlights

• A novel semantic analysis offers insight into the diversity of scientific publications.
• Synthesis center publications are more topically diverse than others.
• Article diversity is strongly and negatively related to a paper's visibility (measured using citations).

Interdisciplinary research, widely heralded as a way to solve complex societal problems and to produce deeply original, even transformative, scientific knowledge, has been pursued and promoted for decades by scientists and science policymakers (National Academy of Sciences 2005; Porter et al., 2006; National Academy of Sciences 2014; Frodeman et al., 2010).^1^ Hopes that interdisciplinary research would arise unassisted through natural processes of random variation and selective retention (Campbell, 1960), consilience (Wilson, 1998), or convergence (Sharp, 2011) have given way to interventions that create organizations and processes to foster interdisciplinary collaboration (see Palmer et al., 2016).^2^ Innovative organizational forms have been designed to promote epistemic integration, ranging from constant co-location of researchers in specifically designed centers or campuses to large-scale networks, such as the European Framework Programmes and COST networks, which bring researchers together over space and time (Biancani et al., 2014, Hackett and Parker, 2016; Barringer et al., n.d.; Vermeulen, 2018).

Recent years have seen prominent and costly investments to build places, organize research, and shape group interactions to facilitate the integration of knowledge across disciplines (Biancani et al., 2019; Kaji-O'Grady et al., 2018; Kleinman et al., 2018; Klonk, 2016; Palmer et al., 2016). Examples include Stanford's Clark Center, which houses Bio-X, and interdisciplinary science and technology buildings on campuses as varied as the Arizona State University, the University of Manchester, and the Humboldt University Berlin. The Howard Hughes Medical Institute built and operates the Janelia Research Campus to embody similar integrative principles and goals, as does the Barcelona Institute of Biomedical Research and the Australian Health Innovation Precincts.

Studies of such research units suggest that different organizational forms, and the social dynamics they promote, lead to different outcomes – whether epistemic (O'Malley & Soyer, 2012), or in terms of interaction patterns (Kabo et al., 2014; Kaplan et al., 2017), industry ties (Leydesdorff and Ivanova, 2020), or article and grant productivity (Biancani et al. 2014). But our knowledge is limited by two characteristics of this body of work. First, it is based largely on case studies of one – or a handful of – centers (exceptions include Boardman and Corley, 2008; Gaughan and Corley, 2010). Second, it rarely examines integration, synthesis, or diversity as an outcome in and of itself (exceptions include Basner et al., 2013 and Biancani et al., 2019). Thus, little is known about how these distinct ways of organizing research affect scientific knowledge. We rectify this imbalance by examining the research products of two synthesis centers and assessing whether they are indeed more diverse than a comparable body of work.

Synthesis centers, perhaps the most visible and potentially effective of such integrative organizations, combine intensive, temporary, co-located collaboration with asynchronous, networked collaboration to achieve epistemic integration (Hampton and Parker, 2011). Beginning with the National Center for Ecological Analysis and Synthesis in 1995, the US National Science Foundation (NSF) has invested in a series of synthesis centers, culminating in the Socio-Ecological Synthesis Center (SESync), the latest and best funded of them.^3^ With their prominence, scale, and apparent success, synthesis centers have been strategic sites for several studies of the process and outcomes of their distinctive form of interdisciplinary collaboration (Hackett et al., 2008; Rhoten and Parker 2003; Hackett and Parker, 2016), but no study yet has asked, *Do synthesis centers synthesize?* That is, if such centers integrate diverse concepts, theories, tools, techniques and data, then the publications of synthesis-center collaborations should be more diverse and, in consequence, more visible than other publications. We address these questions through semantic analysis of the text (e.g., titles, abstracts, and keywords) of published journal articles to compare the topical diversity of publications originating in synthesis centers with publications in a reference corpus of scientific literature.^4^

## 1 What is synthesis?

Scientific synthesis is a form of interdisciplinary research that integrates diverse theories, methods, and data across spatial or temporal scales, scientific phenomena, or forms of expertise to increase the generality, parsimony, applicability, or empirical soundness of scientific explanations (Carpenter et al., 2009; Hackett and Parker, 2016). Synthesis occurs through collaboration among disciplinary or transdisciplinary experts, and therefore encompasses and extends beyond interdisciplinary research. Synthesis counterbalances scientific specialization, capitalizes on existing data, and addresses complex problems (Börner, 2015; Hackett et al., 2008; Palmer et al., 2016). When successful, synthesis draws topics and through them specialties or disciplines together in novel configurations that open new spheres of inquiry and address societal challenges in original and effective ways (Carpenter et al., 2009; Baron et al., 2017; Wyborn et al., 2018).

The first synthesis center, the National Center for Ecological Analysis and Synthesis (NCEAS), was founded in 1995, funded by the US National Science Foundation and the State of California. NCEAS was designed to promote collaborations that extended across academic disciplines and, in some cases, also included environmental policymakers and government officials to address problems of scientific and societal importance (Hackett et al., 2008). In doing so, the center would also transform the practice and outcomes of ecological research. NCEAS's demonstrable accomplishments (through two successful renewals, resulting in more than 15 years of continuous funding), combined with funding agencies’ continued quest for transformative research and solutions to complex practical problems, have resulted in major national and international investments in synthesis. By 2017, nearly two dozen synthesis centers in various fields across the globe are based explicitly on the NCEAS model, representing public investments of many tens of millions of dollars (the US alone has spent about $150M; for a list of centers see e.g., http://synthesis-consortium.org/)

Synthesis centers vary in intellectual foundation and specific aims, but share similar purposes and operating principles, including: (1) a commitment to advance knowledge and address societal challenges through (2) small, self-organized collaborative groups of 6-20 scientists and practitioners (3) drawn from diverse disciplines, professions, and social backgrounds (gender, nationality, seniority) whose work (4) combines spells of intensive, face-to-face collaboration in a setting insulated from day-to-day distraction and routine, separated by longer intervals of remote, computer-mediated work (Hackett et al., 2008; Börner, 2015; Palmer et al., 2016). Synthesis centers explicitly work to fulfill the long-sought promise of interdisciplinary integration (Wilson, 1998; Sharp et al., 2011; National Academy of Sciences, 2014).

Synthesis working groups are formed by a scientific leader who develops a brief proposal to address a compelling scientific research question (often with direct implications for policy or practice, such as evidence-based policies to protect marine mammals or optimized design of a strategy to rescue a depleted fishery) and identifies a group of 6-20 scientists and practitioners with distinctive and complementary expertise to work on the problem. Groups may be formed, led, and composed of scientists from anywhere in the world, and some attention is given to the personal qualities and collaborative propensity of prospective group members. Proposals are competitively reviewed by a science advisory board. Working groups are diverse in composition, often including senior and junior scientists of various disciplines and specialties, as well as resource managers and policy makers. The working group will gather at the center to work intensively for several days on several occasions over a period of 2 to 3 years, with group members remaining in communication with one another and working on aspects of their project during the intervals between meetings.

The immersive intensity of synthesis groups causes a distinct pattern of social interaction that concentrates diverse expertise and promotes cooperation, collegiality, and transdisciplinary collaboration (that extends across academic disciplines to include, for example, government officials and representatives of civic groups). While these are primarily task-oriented groups, because they are immersive they also allow for shared leisure time, which may increase group cohesion and collegiality (Parker and Hackett, 2012; Fine and Corte, 2017). When conservation practice or policy is involved, as happens in about 25% of the groups at NCEAS, the consequences of the research become more visible and salient, lending focus, urgency, and emotional energy to the collaboration (Collins 2004). For example, NCEAS research groups helped develop California's Channel Islands Marine Protected Areas, informed the US Congress about honeybee decline, and studied the ecology of infectious diseases. In such cases the working groups included conservation or environmental policy experts, bringing into the collaboration the local concerns and practical needs of the particular site or problem (for example, species depletion in the Eastern Pacific fisheries or the ongoing stresses experienced by endangered species) and the distinctive perspective of creating knowledge that may provide a basis for intervention.

Synthesis collaborations may promote the creative combination of ideas across disciplines and help to overcome the difficulties that arise when working across institutions (Leahey et al., 2017; Cummings and Kiesler, 2005; Frickel et al., 2017), which are expected to increase the quality and visibility of research that emerges from synthesis centers. Several years of ethnographic observation (Hackett et al., 2008; Hackett and Parker, 2016), quantitative analyses of working group characteristics and performance (Hampton and Parker, 2010), and a pilot study combining sociometric sensors, daily surveys, and ethnography (Parker et al., 2018) showed that synthesis center collaborations produced group characteristics that promote individual and collective creativity (Amabile, 2013; Corte et al., 2019; Parker and Corte, 2017). These characteristics included: (1) resources, both in the form of human expertise and as research material and tools (including bridging social capital); (2) context, removed from everyday status cues and conducive to rich interpersonal interaction though bonding and shared social capital; (3) energy, arising from collective excitement about a motivating research question or compelling societal need (e.g., the use-inspired fundamental research of Pasteur's Quadrant; Stokes 1997); and (4) adaptive management of ambivalence or values in tension.^5^ To illustrate, field observation revealed younger scientists speaking to senior scientists as equals, group bonding rituals and the development of distinctive identities and shared understandings, along with sharply critical interpersonal exchanges (which we called “peer review on the fly”) accelerating the creative process without rending the group, and rapid oscillation from constructive (brainstorming) modes of exchange to critical (evaluative review) of ideas, models, and data (Hackett and Parker, 2016).^6^

Characteristics and dynamics observed in synthesis centers are also found in other contexts that aim to inspire synthesis. For example, Harvey (2014) studied Pixar, the animation studio, and identified many of the same characteristics and dynamics observed in synthesis centers. Among those most conducive to creativity are resources (talent and technology), “a shared understanding that is unique to the collective” that holds the group together (Harvey, 2014: 325), and a process of construction and criticism much like peer review on the fly, in which “group members focus on single ideas in depth, ignore ideas, criticize ideas as they arise, and provide immediate interpersonal rewards for good ideas” (Harvey, 2014: 328). Concepts borrowed from synthesis centers, knowingly or not, inform the interdisciplinary collaborative initiatives of pioneering private foundations and patrons of science, including the Paul G. Allen Family Foundation, the Chan-Zuckerberg Initiative, and the cancer research investments of the Sean Parker Foundation.^7^

Research examining the dynamics and performance of synthesis working groups has found that they spark distinctive and productive forms of social interaction, resulting in highly cited research and enduring career benefits for participants (Hampton and Parker, 2011), yield effective solutions to socio-environmental problems (e.g., design of a successful marine protected area; Lubchenco et al., 2003), increase participants’ propensity to collaborate in the future (Rhoten and Parker, 2004), and enhance the likelihood of serendipitous and potentially transformative research (Hackett et al., 2008; Hackett and Parker, 2016).

Synthesis centers have accelerated the development of collaborative communities, catalyzed research areas, developed novel solutions to vexing societal concerns, and reshaped the social organization and dynamics of research (Rodrigo et al., 2013; Palmer et al., 2016; Baron et al., 2017; Altschul et al., 2017). Despite this body of research, no analysis has yet addressed the fundamental question: *Do synthesis centers synthesize*? Thus, our primary goal is to investigate whether papers from synthesis centers integrate a greater diversity of topics than comparable papers from a reference corpus.^8^ We then assess whether the topical diversity of a publication and its origin in a synthesis center enhance its visibility or impact, as indicated by citations.

## 2 Diversity and synthesis

Diversity is a complex concept that has three principal aspects – variety, evenness, and disparity – each emphasizing a particular quality of the concept (Rao, 1982; Stirling, 2007; Yegros-Yegros et al., 2015).^9^ **Variety** is the number of different items present in a collection of objects or ideas (analogous to “species richness” in ecology): just as a more diverse or varied environment includes a greater number of species, a more diverse or varied publication would include a greater number of topics. **Evenness** is the relative frequency of occurrence of the items in a collection: a more diverse or even publication would include a more uniform (i.e., equal, balanced) distribution of topics. **Disparity** is the degree of difference between items: a more diverse or disparate publication would include topics that are less commonly associated with one another (or found together in a publication) and so are considered more *disparate* from one another. In short, more diverse publications (such as those produced by synthesis center collaborations) may be expected to include a greater variety of topics, a more even distribution of topics, and/or greater disparity between topics (Patil and Taillie, 1982; Stirling, 2007; Yegros-Yegros et al., 2015). Our analysis employs seven conceptually distinct but intrinsically interrelated measures of diversity, either taken as a complex whole or emphasizing one or more of its principal aspect (variety, evenness, and disparity; see Table 1 and Methods, Measures, and Analytic Approach section).

**Table 1:** Diversity concepts, measures, and formulations

Variable	Concept	Mathematical formulation
Variety	Variety	n
Shannon evenness	Evenness	-∑ p_i_ ln(p_i_)/ln(n)
Shannon (entropy)	Variety and Evenness	-∑ p_i_ ln(p_i_)
Mean disparity (Rao-Stirling)	Variety, Evenness and Disparity	-∑_ij_ d_ij_ p_i_ p_j_
Median disparity (Uzzi's conventionality)	Variety, Evenness and Disparity	Value of d_ij_ in the middle of the d_ij_ distribution
Non-weighted disparity	Disparity	-∑_ij_ d_ij_ / [(n-1)*n] (sum only for p_i_ and p_j_ > 0)
Top 10% disparity (Uzzi's novelty)	Disparity (top tail of distribution)	Value of d_ij_ in the 10% of the d_ij_ distribution

Notations: *P_i_* = proportion of paper in topic *i. d*_ij_ = cosine distance between topics *i* and *j, N is total number of topics.*

## 3 Hypotheses

If synthesis centers synthesize, then we expect their publications to be more topically diverse than publications originating in other research environments. Synthesis center working groups are designed to include not only diverse disciplines, but also different sectors (including government and the private sector) and diverse ideas brought by participants to the collaboration. Synthesis centers bring together more fields of knowledge (variety) that are more different from one another (disparity) yet are present in a balanced way (evenness), so we expect their publications also to manifest greater levels than the reference corpus of these components of diversity relative to the reference corpus. This leads to our first hypothesis:

H1 *Synthesis papers display greater topical diversity than papers in the reference corpus*.

Size matters: larger collaborations may have greater breadth and depth, more network connections (social capital), greater credibility (cultural capital), and other advantages like increased productivity, impact (Lee and Bozeman, 2005; Leahey, 2016; Wuchty et al., 2007), and prominence (Peterson et al. 2012). Indeed, “collaborations permit participation in broader research projects” (Abramo et al., 2014). Deliberately assembled to include the breadth of expertise needed for a project, and generally funded well enough to include all necessary participants, synthesis collaborations are likely to be larger than others. By virtue of such qualities, their greater size may make them also more diverse and make their articles more visible. Thus, we also hypothesize that:

H2 *Synthesis collaborations are larger than collaborations in the reference corpus, and their size will have a direct positive effect on diversity and visibility*.

If synthesis collaborations truly differ from other collaborations in quality or character, as shown by the ethnographic studies described above, then such differences should express themselves as differences in diversity (aggregate and dimensions) that are not accounted for by differences in size (measured as numbers of authors, institutions, and references). Thus, we also hypothesize that:

H3 *Size alone does not account for the greater diversity of synthesis center publications.*

Expectations are mixed about the influence of diversity and its dimensions on the visibility of publications and innovations (Fontana, 2018). Research on innovation suggests that information pooled from disparate sources provides a foundation from which new ideas spring (Mednick, 1962; Hargadon, 2002; Fleming and Waguespack, 2007; Corte et al., 2019). In the realm of science, some studies have found that articles and other scientific products (such as patents) that cover diverse topics have greater visibility (Shi et al., 2009; Schilling and Green, 2011; Uzzi et al., 2013; Leahey and Moody, 2014; Lariviere et al., 2015; Lo and Kennedy, 2015; Leahey, Beckman, and Stanko 2017). Other studies suggest an inverted U relationship of visibility with increasing diversity (Larivière and Gingras, 2010; Yegros-Yegros et al., 2015; Fontana et al., 2018). And both Uzzi et al. (2013) and Wang et al. (2015) found more complex relationships with specific forms of diversity (for example, a particular aspect of diversity or a conventional knowledge base with only few atypical combinations) to be most visible.

We contend that the heightened visibility (as gauged by citation counts) of synthesis center papers is not merely a function of the increased audience size that comes from covering more intellectual terrain (Leahey et al., 2017; Leahey & Moody 2014). Rather, papers that bring together and integrate ideas from disparate sources – that *synthesize* ideas – are more valued by the scientific community, and this explains their greater impact (Leahey, 2007). These ideas motivate our final two hypotheses:

H4a: *Diverse papers are more visible than others, even after controlling for collaboration size (authors, institutions, and references) and topic*
H4b: *Synthesis center papers are more visible, even after controlling for diversity and its components (as well as collaboration size and journal impact factor)*

## 4 Methods, measures, and analytic approach

We test these hypotheses using semantic analysis to compare the topical diversity of publications from synthesis centers (which we will call ‘synthesis papers’) with that of a reference corpus drawn from journals in cognate fields and from general science journals (which we will call ‘reference papers’ or the ‘reference corpus’). Doing so focuses the analysis on a measure of the substance or content of publications, rather than on characteristics of authorship groups (which we treat as an upstream property of a collaboration), social organization and dynamics (which we have studied in other work; Hackett and Parker, 2016), intellectual ingredients (measured by the co-occurrence of bibliographic references), productivity, or visibility (using citation-based measures, which we treat as a consequence of collaboration). Synthesis centers are represented by the two centers with the longest operational lives and publication records: NCEAS and the National Evolutionary Synthesis Center (NESCent) (1996-present and 2004-2015, respectively). We analyze words in the titles, abstracts, and keywords of publications to compare the topical diversity of peer-reviewed publications from NCEAS and NESCent with that of a reference corpus of publications representative of these fields (ecology and evolutionary biology, respectively).

We began with all articles published between 1997 and 2013 by scientists working at NCEAS (n=1213), and all articles published between 2004 and 2013 by scientists working at NESCent (n=335). These papers, totaling 1526^10^ in all, were published in 112 different journals, and constitute our set of ‘synthesis papers.’ The published output of working groups was tracked by center administrators using Web of Science, SCOPUS, and direct appeal to all who have participated in a working group.^11^

For comparison, we generated a reference corpus of literature that included 385,566 articles that appeared between 1997 and late 2013 in the 94 top journals (based on eigenfactor scores) for the five disciplinary areas most relevant to research done in NCEAS and NESCent (Ecology, Evolutionary Biology, Biodiversity Conservation, Fisheries, and Forestry). We also included articles from four general science journals (*Science, Nature, PLoS One*, and *PNAS*), and 14 journals that were common outlets for NCEAS and NESCent based research (listed in Appendix A). Metadata for all articles were downloaded from the Web of Science. Given the great difference in size between the set of synthesis papers and the reference corpus, we assess the robustness of our results by replicating all analyses in a smaller, matched sample (see Appendix B). The reanalysis shows the results to be quite robust to this change in the comparison group.

To assess the diversity of ideas present in each article, we first used Latent Dirichlet Allocation (LDA; Blei.et al., 2003; DiMaggio et al., 2013; Griffiths and Steyvers, 2004) to construct topics from the co-occurrence of words contained in the titles, abstracts, and keywords of articles (see Appendix A for methodological details). LDA is an unsupervised probabilistic method of topic modeling that transforms the semantic content of documents into a proportional mixture of topics that is amenable to quantitative analysis. Topic modeling uses observed patterns of term co-occurrence within documents as a basis for probabilistic identification of latent ‘topics,” and then estimates the proportion of each document that is associated with each of the emergent topics. In contrast to classification schemes (such as Web of Science subject categories) or measures derived from an article's bibliography (that is, its “ingredients” or characteristics of the references it cites), topic modeling offers a more detailed measure of the topical content or substance of a published article. LDA's ability to “generat[e] inductively classifications of ideas from texts” (Kaplan and Vakili, 2015) offers a complementary method derived from substantive elements of publications. Synthesis is intended to transform the substance of scientific work—to create original, coherent, integrative knowledge—and so it is worthwhile to look for its effects in the topics represented in a publication.

LDA modeling requires setting initial parameters, such as the number of topics to be formed from the words in the corpus. We experimented with varying numbers of topics—60, 200, and 250—and concluded through independent assessment by a subset of authors that the 200-topic solution (which yielded 152 substantive topics) provided the best balance of refinement (detail) and meaning.^12^ See the Appendix A for examples of topics. Our use of LDA-derived topics as input for diversity measures is novel. To date, most measures of diversity have been based on categories apparent in extant classification schemes, such as the Web of Science's subject categories (Rafols and Meyer, 2010; Leahey et al., 2017), and such measures are usually applied to the bibliographic references of a paper—its ingredients—rather than to the semantic characteristics of the paper itself, which is the approach we take.

Using these topical data, we calculated seven measures of diversity and its dimensions (Table 1). The first two measures – Rao-Stirling diversity (or mean disparity) and conventionality (or median disparity) – are composite measures that include variety (the number of topics in a paper), evenness (the uniformity of the distribution of topics within an article, for a given number of topics), and disparity (the dissimilarity of the topics, given the number of topics).

Recognizing that the various aspects of diversity are intrinsically interrelated, each of the remaining five measures gives greater emphasis to one or two particular aspects. The concept of ‘variety’ is captured by the count of the number of topics represented in a paper. The concept of ‘evenness’ is measured with a normalization of Shannon Entropy, which we call Shannon Evenness, calculated as $-\sum_{i}^{N}P_{i}\ln\left(P_{i}\right)/\ln\left(N\right)$, where *P*_i_ is the weight of each of the *N* topics. We also analyze diversity using the conventional measures of Shannon (entropy), which captures a combination of the concepts of variety and evenness.

Disparity is the most difficult aspect of diversity to measure because doing so requires a prior decision about the salient dimension of difference (Stirling 2014). We calculate a total of four measures of disparity. The first two consider all topics in a given paper and how disparate they are from each other. **Mean disparity**, also known as Rao-Stirling diversity (Rao, 1982; Stirling, 2007), weights the disparity between topics by the relative weight of the topic in the paper. **Median disparity** (based on ‘conventionality’ in Uzzi et al., 2013), is the median value of the disparities across topics in a paper, taking into account the weights of papers.^13^ Since mean and median disparity take into account the full distribution of disparities, they measure disparity in a manner that is implicitly weighted by variety and evenness. In this sense, they are more ‘complete’ or comprehensive measures of diversity because they include all three aspects of diversity (Stirling, 2007).

Two additional measures of disparity aim to isolate disparity from variety. “**Non-weighted disparity**” (Yegros-Yegros et al, 2015) does so by ignoring the proportions (the weights) in the measure of disparity: it is the mean of disparities across topics in a paper, without the proportions. “**Top 10% disparity”** is based on ‘novelty’ in Uzzi et al. (2013). It takes the value of disparity of the 10% of the distribution of disparities across topics in the paper.^14^

We found a small number of very distant outliers in the data, such as publications with more than 100 authors or references, which might bias the analysis. Therefore, for subsequent analyses, we truncated the distributions of addresses and references at the 99^th^ percentile to reduce their distorting influence; this is indicated by a “T” following the variable name. We also controlled for topic (binary variables to capture the 152 topics) and for other potential artifacts of the LDA approach.^15^

To determine whether synthesis papers are not only more diverse but also (perhaps through their ability to synthesize such diversity) more visible, we use a set of conventional measures and control variables. To measure visibility, we use the number of citations a paper accrued as of 2013^16^ and a binary variable indicating whether or not the article is among the top 5% of cited articles. The binary variable focuses the analysis on the question of whether or not a contribution is a “hit” or a major contribution to its field (Uzzi et al., 2013; Lee et al. 2014).

Synthesis collaborations are designed to represent a breadth of scientific expertise and substantive knowledge and have funds to assemble such groups, so they may have more members than others. Larger team size, in turn, brings not only expertise and knowledge but also other forms of human, social, and cultural capital (Collins, 1998; Simonton, 2004; Burt, 2005; Lee et al., 2015; Wuchty et al., 2007; Uzzi et al., 2013; Leahey et al., 2017). For example, meta-analyses conducted by synthesis center groups have twice as many authors, and studied 1.6 times as many species compared to meta-analyses published elsewhere (Cadotte et al., 2012).

We take size into account with three variables: the number of authors of a paper, an indicator of the size of the collaboration; the number of distinct institutions (addresses) represented by authors, which is an indicator of substantive scope and social capital (Burt 2005); and the number of references cited in an article, which is an indicator of the breadth of an article's intellectual foundation (a form of cultural capital; Collins 1998; Simonton, 2004). Each aspect of size – individuals, organizations, and references – is an intellectual resource that may contribute to the diversity and visibility of an article.^17^

We hypothesize that these characteristics of the collaboration not only may account for differences in diversity and visibility, but also may play a mediating role through which properties of synthesis center collaborations influence article diversity and visibility.

Data and code are available from the Dryad Digital Repository (Hackett et al., 2020).

## 5 Results

We expected composite measures of diversity (the Rao-Stirling diversity index and the conventionality index) to be higher for synthesis center papers than for papers in the reference corpus, but found instead that composite diversity measures for synthesis center papers are virtually equal to those of papers in the reference corpus. This unexpected result led us to think more deeply about the varied meanings of diversity (Stirling, 2007) and to include in the analysis measures that emphasize one or another dimension of diversity; namely, variety, evenness, and disparity. Comparing mean values for each of the three measures yields a more precise result: synthesis center papers have greater topical variety and evenness (or balance) than papers in the reference corpus, partially supporting Hypothesis 1 (although the mean differences are small). However, the topics that characterize synthesis center papers are less disparate than those derived from the reference corpus.

We then asked if the greater topical variety and evenness of synthesis papers is the result of their having more authors, more references, or a greater number of distinct institutions than have papers in the reference corpus? More authors, institutions, and references would bring a greater breadth of social and intellectual resources (capital) to collaborations. We find that, on average, synthesis center papers have slightly (but not significantly) more authors than papers in the reference corpus, and significantly greater numbers of distinct institutional affiliations and references (see Table 3), lending partial support to Hypothesis 2. Even when year of publication and modal topic are controlled (Table 4), synthesis center papers have significantly more institutions represented in their publications and cite more literature than do papers in the reference corpus. However, contrary to Hypothesis 3, we find that papers with more authors are not more diverse (see Table 5): in fact, on all measures, a larger authorship group is associated with less diversity (Bernstein et al., 2018). Thus, while synthesis center collaborations have greater variety and evenness, this effect cannot be explained by their larger size (i.e., numbers of authors, institutions, and references). Collaboration size and the advantages it brings do not mediate the relationship between synthesis center affiliation and diversity. Even after controlling for other variables^18^, the differences in Table 2 remain: publications of synthesis center collaborations have greater variety and balance, but less disparity, than papers in the reference corpus (Table 5). The effect of synthesis center affiliation is substantial: its effect on variety is equivalent to adding six authors to a paper (6 x .034), and its effect on evenness is equivalent to adding ten institutions (based on comparing the coefficient of the synthetic dummy variable with the coefficients on the authors and institutions variables).

**Table 2:** Mean differences in diversity and its components, synthesis centers and reference corpus (N=398,378).

	Variable	Synthesis center articles (n=1526)	Reference corpus (n=396,852)	Difference (sig.)
Variety	Variety	6.75	6.18	.578***
Evenness	Shannon Evenness	0.855	0.812	.043***
Shannon	Shannon Entropy	1.96	0.79	0.168***
Stirling	Mean Disparity	0.33	0.34	0.004
D50	Median Disparity	0.35	0.36	0.007
Disparity	Non-weighted Disparity	0.417	0.461	-.043***
D90	Top 10% Disparity	0.82	0.84	-.019***

As hypothesized (Hypothesis 4b), synthesis center papers receive more citations than papers in the reference corpus. Table 6 shows that the differences are substantial: twice as many citations and twice the probability of being among the top 5% of all articles (“hits” or very visible articles). But recall that synthesis collaborations are larger in some respects (institutions, references; see Table 5) and the publications they produce are more diverse (in terms of variety and evenness) and less disparate than those of the reference corpus, so it is necessary to consider size and diversity, and to include other variables, to determine whether (and the means by which) synthesis collaborations produce more visible publications.

These differences in visibility are not explained by group size or diversity. To determine this, we modeled two visibility outcome variables: 1) the number of citations, using negative binomial regression for count data, and 2) the binary property of a paper being a “hit” (in the top 5% of the citation distribution) or not, using logistic regression analysis. Control variables in each model include size and heterogeneity of the collaborative group (numbers of authors, institutions, and references), synthesis center affiliation, and one of the seven diversity variables, as well as a set of technical control variables (listed in the Table 7 footnote). Table 7 shows that the greater the size and heterogeneity of a collaborative group associated with a publication, the greater the number of citations it will receive and the greater the likelihood that it will be among the top 5% of the citation distribution (a “hit”). Taking that into account, synthesis center origins retain a strong, positive effect on both visibility measures, as well as an indirect effect mediated by size and heterogeneity. Finally, with all that taken into account, every measure of diversity—both aggregate and facet—has a negative effect on citations and on the likelihood of a “hit.”

Given previous literature, we hypothesized that diversity and related measures would have positive effects on both measures of visibility, and were surprised to find that diversity and related measures have significant *negative* effects on both measures of visibility. We acknowledge that the difference may be a consequence of our reliance on categories derived from topic modeling (rather than, say, Web of Science subject categories), particular control variables used in the models, mediation by measures of collaborative groups (authors, institutions, and references), or other such differences in method or model specification. We also acknowledge that citation-based indicators may under-represent the visibility of interdisciplinary publications and that other visibility measures should be developed (Ràfols et al., 2012). That said, this result suggests that there are unmeasured characteristics of synthesis publications that are associated with increased visibility.

Our comparison of synthesis center papers and a reference corpus reveals that synthesis center papers are slightly more diverse (in terms of the variety of topics integrated, and the evenness of those topics), and that this effect is only partly mediated by the greater resources (numbers of authors, distinct institutions, and references –Table 3) of synthesis center collaborations.  But even when taking size and heterogeneity into account, there is a persistent direct effect of synthesis centers, suggesting that synthesis center collaborations benefit from a distinctive quality that is not measured in this study.

**Table 3:** Mean differences in collaboration and publication characteristics, synthesis center articles vs. reference corpus.

Variable	Synthesis center articles (n=1526)	Reference corpus (n=396,852)	Difference (t)
Number of authors	5.19	4.92	.27 (.23)
Number of addresses	4.48	2.81	1.66 (22.2)***
Number of references	56	44.4	11.6 (21.8)***
Proportion of topic weight removed as junk	0.354	0.334	.021 (8.31)***
Count of tokens per document (after stop word removal)	109.3	111.3	2.00 (2.35)
Publication year	2006	2006.8	.737 (6.03)***

* = p < .05; ** = p < .01; *** = p < .001

Several factors may be responsible for these effects. Synthesis center collaborations include extended periods of intense and isolated face-to-face interactions, which may build trust, increase emotional energy, and give rise to a host of beneficial social dynamics that sustain the group through periods of distal collaboration (Collins 1998; Farrell, 2001; Hackett and Parker, 2016). Recurrent face-to-face meetings also afford groups time to establish and maintain boundaries and to surface and resolve the epistemological, methodological, and substantive differences that invariably arise in diverse teams (Bernstein et al., 2018; Boix Mansilla et al., 2016; Parker and Hackett, 2012; Penders et al., 2008).

However, synthesis center papers are not more disparate than papers in the reference corpus. That is, although they incorporate more topics in a more balanced way, the topics themselves are not more disparate or distant in cognitive space from one another. Synthesis center collaborations are certainly inter- or transdisciplinary, working with and integrating topics that hang together well; but they are not pulling together wildly different topics. Perhaps one feature of synthesis center collaborations is that they balance the novelty that comes from joining disparate topics with the conventionality that comes from working within the framework of topics that are similar to one another and readily joined together (Uzzi et al., 2013; Frenette, 2019).

Although it is consistent with some recent work (Wu, Wang, and Evans 2019), we did not expect team size to be associated with lower levels of diversity. If the main purpose of collaboration is to pool specialized knowledge (Maienschein, 1993; Hackett, 2005), then papers with more authors should have greater diversity. But perhaps pooling knowledge is not the dominant motivation for collaboration (Leahey and Reikowsky 2008; Leahey, 2016). Perhaps collaboration adds person-power to accomplish more quickly a shared set of similar tasks, rather than to complete a differentiated set of dissimilar tasks. Or, perhaps, a topic (in the sense of this paper) is broader than one scientist's expertise and so two or more scientists may be needed to accomplish the work represented by a topic. And, finally, the causal arrow may run in the opposite direction: perhaps a substantial degree of intellectual, interpersonal, or technical *similarity* (such as shared use of a research tool or system) is necessary to sustain and hold together a collaboration with many members (cf. Farrell, 2001; Parker and Corte, 2017), and, in the absence of such conditions, collaborations remain small. Components of the complex concept “size” and components of the complex concept “diversity” may have distinctive relationships with one another. For example, Table 4 shows that the number of institutions in a collaboration significantly increases overall diversity and all its components, but that the number of references (an indicator of size that emphasizes intellectual or cultural capital) increases two aspects of diversity–variety and balance—but not the third (disparity).

**Table 4:** OLS regression of numbers of authors, institutions, and references on synthesis center affiliation and control variables.

	# Authors	# Institutions	# References/10	# InstitutionsT	# ReferencesT
Synthesis center paper (1=yes) (95% confidence interval)	.870 (.75-.98)	1.39 (1.29-1.50)	.777 (.68-.87)	1.01 (.91-1.10)	.775*** (.68-.87)
Number of Authors	–	–	–	.453	.004**
Constant	2.86	4.01	5.62	2.67	5.6
R^2^ adj	0.27	0.13	0.15	0.37	0.15
N	396,852	396,852	396,852	396,852	396,852

Note: Although coefficients are not shown, we also controlled for year of publication and modal topic (indicator variables for 152 topics). “T” = truncated, “/10” = divided by 10 to rescale, 95% confidence intervals for synthesis dummy variable are in parentheses.

* = p < .05; ** = p < .01; *** = p < .001

**Table 5:** OLS regression of diversity measures on synthesis center origin, controlling for team characteristics.

	Variety	Shannon Evenness	Shannon Entropy	Mean Disparity	Median Disparity	Non-weighted Disparity	Top 10% Disparity
Synthesis center paper (1 = yes)	0.198***	0.014***	0.0450***	.006*	0.009*	-.009**	-0.004*
(95% confidence interval)	(.10-.30)	(.01-.02)	(.03-.07)	(.00-.01)	(.00-.02)	(-0.02–0.00)	(-0.01–.00)
AuthorsT	-0.034***	-0.001***	-0.003***	-.001***	-0.002***	-.001***	0.000
InstitutionsT	0.034***	0.001***	0.005***	0.002***	0.002***	0.001***	0.001***
References/10	0.020***	0.002***	0.009***	-0.001***	-0.001***	-0.004***	-0.000***
Constant	8.82***	0.72***		0.42***	0.37***	0.64***	
R^2^	0.015	0.17	0.19	0.19	0.17	0.28	0.17
N	398,377	398,377	398,377	398,377	398,377	398,377	398,377

Note: All models also control for: year of publication; topic weight removed, number of tokens, and modal topic (dummy variable with 152 categories).

“T”=truncated, “/10”=divided by 10, * = p < .05; ** = p < .01; *** = p < .001

Synthesis center papers are more visible than papers in the reference corpus (Table 6), and such differences are mediated, in part, by size and dimensions of diversity (Table 7). While such qualities of collaboration partly account for differences between synthesis papers and the reference corpus, with such variables controlled synthesis center papers still have significantly (and substantially) greater citation counts than papers in the reference corpus (Table 7).  Synthesis center effects are mediated, to some degree, by collaboration size and the dimensions of diversity. But the strong and significant positive effect of synthesis centers on citation counts and on the likelihood of being a “hit” paper are robust and enduring.

**Table 6:** Differences in visibility for synthesis center articles and reference corpus.

Variable	Synthesis center articles (n=1526)	Reference corpus (n=396,852)	Difference
# Citations received as of 2013	82.7	42.9	*t*-test: 41.6 (11.9)***
% articles that are "hits" (in top 5% of citation distribution)	12.70%	5.90%	χ^2^: 128.9***

Significance levels indicated by asterisks: *= p < .05; **= p < .01; *** = p < .001.

**Table 7:** Regression of article visibility on synthesis center affiliation, diversity measures, and team characteristics.

Panel A. Number of Citations as of 2013							

Synthesis center paper (1 = yes)	0.513***	0.511***	0.512***	0.509***	0.508***	0.504***	0.504***
(95% confidence interval)	(0.47-0.56)	(.46-.56)	(.46-.55)	(.47-.56)	(.46-.55)	(.46-.55)	(.46-.54)
AuthorsT	0.064***	0.065***	0.064***	0.064***	0.064***	0.065***	0.065***
InstitutionsT	0.014***	0.013***	0.014***	0.014***	0.013***	0.013***	0.013***
References10	0.117***	0.117***	0.12***	0.116***	0.116***	0.116***	0.116***
Variety	-0.025***						
Shannon Evenness		-0.246***					
Shannon			-0.10***				
Mean Disparity				-0.440***			
Median Disparity					-0.237***		
Non-weighted Disparity						-0.261***	
Top 10% Disparity							-0.286***
Constant (alpha)	3.517***	3.501***	3.490***	3.499***	3.404***	3.503***	3.557***
N	398,358	398,358	398,358	398,358	398,358	398,358	398,358
R^2^ (pseudo for cites)			0.15				

Panel B. Top 5% of Citation Distribution							

Synthesis center paper (1 = yes )	0.977***	0.976***	0.973***	0.962***	0.962***	0.953***	0.955***
(95% confidence interval)	(.72-1.1)	(.72-1.1)	(.71-1.1)	(.70-1.1)	(.70-1.1)	(.69-1.1)	(.69-1.1)
AuthorsT	0.167***	0.169***	0.170***	0.167***	0.167***	0.168***	0.169***
InstitutionsT	0.016***	0.015***	0.013**	0.016***	0.016***	0.014**	0.014**
References10	0.265***	0.266***	0.264***	0.265***	0.264***	0.264***	0.264***
Variety	-0.057***						
Shannon Evenness		-0.951***					
Shannon Entropy			-0.217***				
Mean Disparity				-1.221***			
Median Disparity					-0.712***		
Non-weighted Disparity						-0.543***	
Top 10% Disparity							-0.92***
Constant (alpha)	-3.498***	-3.312***	-3.605***	-3.470***	-3.741***	-3.643***	-3.239***
N	398,358	398,358	398,358	398,358	398,358	398,358	398,358
R^2^ (pseudo for cites)	0.46	0.47	0.47	0.47	0.47	0.46	0.47

Note: All models also control for: year of publication; journal influence score, topic weight removed, number of tokens, and modal topic (dummy variable with 152 categories); 95% confidence intervals are in parentheses.

## 6 Conclusion

Scientific synthesis has arisen rapidly in response to challenges such as overcoming hyper-specialization, navigating immense and growing literatures, conceptualizing complex socio-environmental problems, and enhancing the potential for serendipitous discovery and transformative research. Synthesis is essential in a world where scientific specialists must collaborate to solve complex intellectual puzzles and ‘wicked’ practical problems that lie beyond the reach of any one discipline, profession, dataset, method, or theory. Research has shown the synthesis working group to be a distinctive form of scientific collaboration that reliably produces consequential, high-impact publications, but no one has attempted to directly investigate their *raison d'être*: do synthesis working groups produce publications that are substantially more diverse than those produced outside of synthesis centers, and if so, how and with what effects? We have investigated these questions through a novel textual analysis. Let us emphasize the originality of the approach: We are not sure how measuring diversity in terms of topics obtained from topic modelling rather than from co-citation, bibliographic coupling, Web of Science categories, or other bibliometric means differ, though we do know that such measures often disagree with one another, thus generating fruitful leads to better understanding (Wang and Schneider, 2018). We have not tested the measure by validating it against other properties of specific articles or researchers, but we do know that it taps into the substance of the articles—meaningful words—rather than more distal properties. The power and robustness of the measure remain to be determined, yet we think it has shown sufficient promise to merit further investigation as a complement to bibliometric approaches.

What have we learned? Overall, synthesis center publications have greater numbers of authors from more institutions than do publications in the reference corpus, and these integrate a broader conceptual and knowledge base, as measured by numbers of references. Surprisingly, having a greater number of authors is not associated with greater topical diversity, but having a greater number of distinct institutional addresses is. Papers with more authors and more institutions are also more highly cited. Article diversity, as a whole or in components, is strongly and negatively related to citation counts and the probability of being a hit paper (i.e., falling within the top 5% of the citation distribution). Synthesis papers are slightly more topically varied and balanced, and despite this, are highly cited, suggesting that unmeasured properties of synthesis center publications are relevant to their impact^19^.

Our research also yields several practical lessons. First, the positive association of synthesis center papers with diversity, citations, and influence suggests that despite the current excitement and necessity around ‘virtual organizations’ and distal forms of collaboration, there is still a place for physical centers and face-to-face groups. They appear to be effective arrangements for produccing transformative and synthetic research. Second, policies intended to identify and support transformative research (NSB 2008) have attempted to do so by selecting particularly promising projects or people, generally with very low award rates. This study suggests that there is merit in creating organizations, such as synthesis centers, that integrate diverse concepts, methods, and data. Third, text analysis is a rapidly evolving field with substantial promise for revealing the substance and intellectual dynamics of science, complementing bibliometric measures of scientific properties and performance. Finally, current demands for transformative scientific knowledge and innovative solutions to pressing practical problems have stimulated policy and programmatic interest in convergence (Sharp et al., 2011; NAS, 2014). Such organizational innovations are in their infancy and should be regarded as experiments, informed and adaptively managed by analyses of their collaborative processes and research outcomes.

## CRediT authorship contribution statement

**Edward J. Hackett:** Conceptualization, Funding acquisition, Writing - review & editing. **Erin Leahey:** Conceptualization, Methodology, Formal analysis. **John N. Parker:** Conceptualization, Methodology, Writing - review & editing. **Ismael Rafols:** Conceptualization, Methodology, Formal analysis. **Stephanie E. Hampton:** Conceptualization, Funding acquisition, Writing - review & editing. **Ugo Corte:** Conceptualization, Writing - review & editing. **Diego Chavarro:** Methodology, Formal analysis. **John M. Drake:** Methodology, Formal analysis. **Bart Penders:** Conceptualization, Writing - review & editing. **Laura Sheble:** Conceptualization, Writing - review & editing. **Niki Vermeulen:** Conceptualization, Writing - review & editing. **Todd J. Vision:** Conceptualization, Writing - review & editing.

## Declaration of Competing Interest

The authors declare that they have no known competing financial interests or personal relationships that could have appeared to influence the work reported in this paper.

## Figures and Tables

**Table A1:** Mean differences in diversity and its components, synthesis centers and reference corpus (N=15,037) matched sample (up to 10 controls per synthesis center paper)

	Variable	Synthesis center articles (n=1526)	Reference corpus (n=13,535)	Difference (sig.)
Variety	Variety	6.75	6.41	0.034***
Evenness	Shannon Evenness	0.85	0.83	0.026***
Shannon	Shannon Entropy	1.96	1.85	0.106***
Stirling	Mean Disparity	0.33	0.33	-0.002
D50	Median Disparity	0.35	0.35	0.000
Disparity	Non-weighted Disparity	0.42	0.44	0.02
D90	Top 10% Variety	0.83	0.83	0.008

**Table A2:** Mean differences in collaboration and publication characteristics, synthesis center articles vs. reference corpus

Variable	Synthesis center articles (n=1526)	Reference corpus (n=13,535)	Difference (sig.)
Number of authors	4.80	4.31	0.49***
Number of institutions	4.24	2.66	1.58***
Number of references	55.7	48.4	7.43***
Proportion of topic weight removed as junk	0.35	0.34	0.012***
Count of tokens per document (after stop word removal)	109	113	4.3***
Publication year	2006	2006	0.032

*= p < .05; **= p < .01; ***= p < .001

**Table A3:** OLS regression of numbers of authors, institutions, and references on synthesis center affiliation and control variables

	# Authors	# Institutions	# References/10	# InstitutionsT	# ReferencesT
Synthesis center paper (1=yes)	0.55***	1.23***	0.54***	0.93***	0.52***
Number of Authors	–	–	–	0.54***	0.04***
Constant	2.48***	3.53***	6.29***	2.18***	6.19***
R^2^ adj	0.16	0.14	0.12	0.51	0.12
N	15,037	15,037	15,037	15,037	15,037

Note: Although coefficients are not shown, we also controlled for year of publication and modal topic (indicator variables for 152 topics). "T"=truncated, "/10"=divided by 10.

* = p < .05; ** = p < .01; *** = p < .001

**Table A4:** OLS regression of diversity measures on synthesis center origin, controlling for team characteristics.

	Variety	Shannon Evenness	Shannon Entropy	Mean Disparity	Median Disparity	Non-weighted Disparity	Top 10% Disparity
Synthesis center paper (1 = yes)	0.202***	0.015***	0.053***	0.010***	0.015**	-0.002	-0.001
AuthorsT	-0.040***	-0.002***	-0.005**	-0.002***	-0.003***	-0.002**	0
InstitutionsT	0.038***	0.002**	0.006**	0.002**	0.002*	0.001	0.001*
References/10	0.018*	0.001**	0.007***	-0.002***	-0.002**	-0.004***	-0.001***
Constant	7.900***	0.729***	1.662***	0.344***	0.254***	0.548***	0.798***
R^2^	0.13	0.13	0.14	0.20	0.18	0.13	0.17
N	15,037	15,037	15,037	15,037	15,037	15,037	15,037

**Table A5:** Differences in visibility for synthesis center articles and reference corpus.

Variable	Synthesis center articles (n=1502)	Reference corpus (n=13,535)	Difference
# Citations received as of 2013	79.9	40.3	39.65***
% articles that are "hits" (in top 5% of citation distribution)	12.25%	4.26%	X^2^: 128.9***

Significance levels indicated by asterisks: *= p < .05; **= p < .01; *** = p < .001

**Table A6:** Regression of article visibility on synthesis center affiliation, diversity measures, and team characteristics

Panel A. # Citations as of 2013							

Synthesis center paper (1 = yes)	0.440***	0.436***	0.431***	0.441***	0.438***	0.433***	0.431***
AuthorsT	0.036***	0.036***	0.036***	0.036***	0.036***	0.036***	0.037***
InstitutionsT	0.029***	0.029***	0.030***	0.029***	0.029***	0.028***	0.029***
References10	0.123***	0.123***	0.123***	0.122***	0.123***	0.121***	0.122***
Variety	-0.025***						
Shannon Evenness		-0.233***					
Shannon			-0.104***				
Mean Disparity				-0.585***			
Median Disparity					-0.305***		
Non-weighted Disparity						-0.445***	
Top 10% Disparity							-0.737***
Constant (alpha)	7.900***	0.729***	15.276*	0.344***	0.254***	0.548***	0.798***
N	15,037	15,011	15,037	15,037	15,037	15,011	15,037
pseudo R^2^	0.12	0.12	0.12	0.12	0.12	0.12	0.12

Panel B. Top 5% of Citation Distribution							

Synthesis center paper (1 = yes)	0.962***	0.948***	0.922***	0.953***	0.953***	0.919***	0.925***
AuthorsT	0.139***	0.145***	0.144***	0.137***	0.138***	0.143***	0.143***
InstitutionsT	0.022	0.019	0.017	0.022	0.022	0.018	0.018
References10	0.335***	0.335***	0.333***	0.333***	0.333***	0.331***	0.332***
Variety	-0.097***						
Shannon Evenness		-1.199**					
Shannon			-0.322**				
Mean Disparity				-1.973***			
Median Disparity					-1.295***		
Non-weighted Disparity						-1.012**	
Top 10% Disparity							-2.806***
Constant (alpha)	-3.126***	-3.051***	-3.986***	-3.219***	-3.589***	-3.377***	-1.724*
N	12469	12439	12469	12469	12469	12439	12469
pseudo R^2^	0.44	0.43	0.43	0.44	0.43	0.43	0.44

Note: All models also control for: year of publication; journal influence score, topic weight removed, number of tokens, and modal topic (dummy variable with 151 categories).

Significance levels indicated by asterisks: *= p < .05; **= p < .01; ***= p< .001

**Table:** 

	variety	ShanEven	Shannon	MeanDisp	MedDisp	NWDisp	Top10Disp
Variety	1.0000						
Evenness	0.0148	1.0000					
Shannon	0.8181	0.0387	1.0000				
Stirling	0.6397	0.0274	0.7169	1.0000			
D50	0.4320	0.0263	0.5943	0.8830	1.0000		
Disparity	0.3647	-0.0077	0.1533	0.5794	0.3901	1.0000	
D90	0.2438	0.0032	0.2098	0.4869	0.4031	0.5673	1.0000

**Table:** 

	synth	year	cites	top5	numauthT	numaddT	numre~10
Synth	1.0000						
Year	-0.0048	1.0000					
Cites	0.0177	-0.2476	1.0000				
top5	0.0180	-0.2443	0.5891	1.0000			
numauthT	-0.0012	0.2796	0.0688	0.0993	1.0000		
numaddT	0.0300	0.0965	0.0408	0.0500	0.5031	1.0000	
numrefs10	0.0352	0.1647	-0.0326	-0.0597	-0.0046	0.0071	1.0000

## Appendix B Matched sample

Given the large imbalance between the 1526 synthesis center papers and the hundreds of thousands of papers in the reference corpus, we decided to use matching to select a small number of ‘control cases’ for each synthesis center paper (‘treatment case’). We implemented exact matching in Stata15 using the ‘joinby’ command, by identifying all control cases that share three characteristics with each given treatment case: subject area; number of authors; and year of publication. From these possibilities, up to 10 control cases were randomly selected; if a given treatment case had fewer than 10 possible controls, all were selected. This resulted in a more balanced sample of papers, consisting of 1526 synthesis center papers and 15,535 matched controls. We re-ran all analyses using this smaller sample, and the results (presented in Tables A1, A2, A3, A4, A5,-A6) do not change substantively.

## Appendix C Correlations

Correlations among diversity measures (N=398,378)

Correlations among other variables (N = 398,378)

## Appendix D Algorithm to produce Top 10% disparity and median disparity

Median disparity is the equivalent of Uzzi's conventionality measure. In our adaptation, median disparity is the distance between topics, taken from the distance matrix, when the cumulative sum of articles' weights reaches 0.5. In our script this variable is labeled D50.

Top 10% disparity is the equivalent of Uzzi's “novelty" measure. In our adaptation, Top 10% disparity is the distance between topics, taken from the distance matrix, when the cumulative sum of article's weights reaches 0.9. In our script this variable is labeled D90.

In order to calculate D50 and D90 the distance matrix was sorted in ascending order, this order was matched with the article's weights between topics, and then the cumulative sum of articles' weights was calculated. The algorithm is as follows:

1 Sort in ascending order the distance matrix according to the distance between topics
2 For each article:
2.3 Assign its weights to their corresponding position in the distance matrix.
2.4 Calculate the cumulative sum of each article's weights.
2.5 Find the values at which the cumulative sum reaches 0.5 and 0.9
2.6 Select the distance between topics (from the distance matrix) at the intersection between these two points. These correspond to D50 and D90 respectively.

## Appendix D Supplementary materials

Image, application 1

## Footnotes

1 Following other analyses of interdisciplinarity, ours is “based on the concept of integration: a mode of research that integrates concepts or theories, tools or techniques, information or data from different bodies of knowledge” (NRC 2005; Yegros-Yegros et al. 2015: 7). This definition is more than convenient: it also invokes the conceptualization of creativity as grounded in the association of different ideas (Mednick 1962; Amabile 1983; Simonton 2004).

2 Consilience, a process of “jumping together” (jumping is the “*siliens*” part, as in resilience), proposes that diverse fields of knowledge—not just sciences but also humanities and social sciences—would jump together through an almost elective affinity to address complex societal and intellectual problems with broader, deeper, and more fundamental (some might say mechanistic, even bio-reductionist) explanations. Convergence asserts that certain fields are bending, turning, tending toward one another (the literal meaning of the Latin root *verger)*, and perhaps need some assistance (or removal of resistance) to accelerate the process. For example, an MIT report (Sharp, 2011) argued for investing in the convergence of the life sciences and engineering to bring fundamental knowledge coupled with know-how to bear on health needs (and, reciprocally, to use health needs to inspire fundamental research). NAS (2014) issued a report on convergence, and convergence is among the 10 Big Ideas guiding NSF investments (https://www.nsf.gov/news/special_reports/big_ideas/).

3 Other US centers focused on evolutionary science and mathematical biology. About 24 synthesis centers have been developed worldwide, across all fields of science. By mid-2019 NSF had awarded about $150M to build and operate five synthesis centers (NCEAS, NESCent, NIMBios, SESync, and CIDER-II (for geosciences; figures from award search of nsf.gov).

4 Other analyses use the co-occurrence of subject matter categories of the references in a paper to measure its diversity (Porter et al. 2007; Uzzi et al., 2013; Yegros-Yegros et al., 2015). We think the words in the title, abstract, and keywords offer a complementary view of interdisciplinarity that is based on the output of research rather than the ingredients. Our approach is also less sensitive than others to the politics of classification.

5 Ethnographic studies of synthesis center collaborations identify several characteristics that might enhance interdisciplinary integration (Rhoten 2003; Hackett et al. 2008; Hackett and Parker 2016; Parker et al. 2018). Synthesis centers host concentrated collaborations in settings free from outside distractions and many of the usual marks of status (e.g., professor, student). Their small size and intense, immersive group dynamics mean that collaborators engage one another both as intellects and as whole persons. In turn, these qualities of group structure and interaction reduce status differences, balanced participation, accelerate communication, and sustain trust, which allow ideas to be rapidly proposed, evaluated, and revised (Wooley et al. 2010; Hackett and Parker 2016; Bernstein et al. 2018). Synthesis centers are also resource-rich environments with full-time administrative and technical staff, resident researchers, and access to state-of-the-art computer software and hardware. Finally, synthesis centers instill a commitment to excellence among group members. They are ‘evocative environments’– places known to produce consequential research, challenging and motivating working group members to produce research of equal or greater quality (Zuckerman 1977). These are all beneficial aspects of synthesis centers and working group processes that are unlikely to be replicated in more traditional research environments, and which may help explain the remaining impact and influence associated with a paper originating in a synthesis center. Finally, synthesis centers may promote scientific innovation by increasing the urgency and visible consequences of research (Gordon and Marquis 1966).

6 Social capital is created through the connections a person has with others. Bonding social capital is formed in intensive interactions, which bridging social capital arises through broad, diverse interpersonal connections (Paxton, 1999).

7 https://www.alleninstitute.org/what-we-do/frontiers-group/; http://www.parker.org/about;

8 Unlike measures of interdisciplinarity that are applied to the bibliographic references of an article—its ingredients–topical diversity is an indicator applied to an intellectual product (in this case a published article).

9 Rao-Stirling is one of a family of diversity measures, known as Leinster-Cobbold diversity (Mugabushaka, Kyriakou, and Papazoglou, 2016).

10 A few papers (n=22) had authors from both synthesis centers. These papers were included only once.

11 Center administrators are quite thorough in searching for publications and quite energetic in soliciting self-reported publications because such measures are reported annually to sponsoring agencies. We have been on the receiving end of such attention since 2001.

12 LDA is substantively naïve and so, along with meaningful topics the method also creates a small number of topics that convey little substantive meaning about the paper, such as those formed by the co-occurrence of numeral (one, two, three), directional (up, down), and comparative (greater, lesser) terms. When such terms co-occur they create an apparent topic with no clear substantive meaning that we treated as a data artifact, as is usual practice (Kaplan and Vakili 2014). We removed such terms from the analysis and used the fraction of a topic's weight that was removed in this fashion as a control variable in our analysis to account for any effect this may have had on outcomes of interest here. For similar reasons we also controlled for differences in the number of valid single or multi-word terms for each paper. Weighted sets of representative terms for each topic give substantive meaning to topics, and a solution that yielded 152 substantive topics (and 46 meaningless topics) was judged most representative of the substance of the papers.

13 This is computed as follows. Order the disparities between topics in ascending order from zero to one. Assign a proportion of *P_i_P_j_* to each combination of topics. Pick the value of disparity at which the cumulative sum of proportion reaches 0.5: the median.

14 This is computed as follows. Order the disparities between topics in ascending order from zero to one. Assign a proportion of p_i_p_j_ to each combination of topics. Pick the value of disparity at which the cumulative sum of proportion reaches 0.9 – the top 10%. See Appendix D for details.

15 These other control variables are percent of topics removed and number of tokens used to characterize an article.

16 We recognize that citations are not always positive (see MacRoberts and MacRoberts 1996). However, citations to work indicate its usefulness and provide visibility in the scientific community – both of which signal impact.

17 The numbers of authors and institutions are moderately correlated (*r*=0.645); the number of references is correlated neither with the number of authors nor the number of institutions (Appendix C).

18 Other qualities of collaborations that we have not measured here (but have studied with other methods and reported elsewhere; see Hackett and Parker 2016; Hampton and Parker 2011) may also influence diversity. For example, how much group members have worked together outside this particular collaboration, or group leaders’ selection biases.

19 Mäkinen et al., 2019 show how interdisciplinary research centers’ missions, physical architectures, leadership and task structures affect collaborators’ motivation, interaction, and inclusion, in turn shaping their contributions to interdisciplinary knowledge. Hollingsworth and Hollingsworth (2000) noted similar findings. These unmeasured aspects of synthesis centers may also explain some of the variance in topical diversity and publication visibility that remains unaccounted for in our regression models.
