# Supplementary material for: Do synthesis centers synthesize? A semantic analysis of topical diversity in research
Source: Res Policy. 2021 Jan;50(1):104069. doi: 10.1016/j.respol.2020.104069 (PMC7695893; doi:10.1016/j.respol.2020.104069)
Supplement: Supplementary file 1 [file mmc1.docx]

**Appendix A. Supplemental Methodological Information**

**Topic model**

We created and analyzed a corpus of 398,444 academic journal articles published between 1996 and September 2013 in the fields of Biodiversity Conservation, Ecology, Evolutionary Biology, Fisheries and Forestry. A few document records from 1994 and 1995 that contained abstracts were also included. Publication records were retrieved from Web of Science for the top 20 (based on eigenfactor scores^[[1]](#footnote-1)^) journals in the 5 disciplinary areas, for 4 high-visibility journals, and for 14 journals that are important publication venues for two synthesis research centers. Topic models were generated by applying Latent Dirichlet Allocation to a file containing titles, abstracts, and keywords for these articles. Data include basic information about the documents modeled (e.g., year of publication, number of authors), estimates of the proportion of each document that is associated with each of the emergent topics, and representative terms that allow conceptual interpretation of the topics.[1]

*Document sources*

Documents included in this corpus are from scientific journals that may be grouped into three categories:

• 4 high-visibility general science journals (Science, Nature, PLOSONE, and Proceedings of the National Academy of Sciences (US)).

• 20 leading disciplinary journals (based on 2011 eigenfactor rankings) for each of 5 subject areas relevant to the synthesis centers we studied: ***Biodiversity Conservation***, ***Ecology***, ***Evolutionary Biology***, ***Fisheries***, and ***Forestry.*** This yielded 94 journals, not 100, because in several cases the same journal was among the top 20 for more than one subject area.

• 14 journals that are common publication venues for the two research centers of interest:the National Center for Ecological Analysis and Synthesis (NCEAS) at UC Santa

Barbara, www.nceas.ucsb.edu; and the National Evolutionary Synthesis Center

(NESCent) at Duke University, UNC Chapel Hill and NCSU, www.nescent.org.

*Document characteristics – manifest*

Information about journal category (as described above) and year of publication is included for each document. See more detailed descriptions in the list variables below.

*Document characteristics – derived*

Two quantitative variables that indicate the number of authors and number of unique author addresses for each document were created through mining of the original qualitative document data. The number of word tokens in the document (after preprocessing and stop word removal; see pre-processing, below) was also calculated and saved as a variable. Derived variables are described below.

*Document preprocessing*

Documents were pre-processed to remove or replace symbols and special characters, and to remove journal-specific subheadings within abstracts (e.g., ‘Motivations’, ‘Background’, ‘Methods’), citations within abstracts, and copyright statements. All instances of a set of ‘stop words’ and terms that appeared only once throughout the entire corpus were removed prior to topic modeling to reduce computational intensity and improve the quality and interpretability of the resulting model outputs.

*Topic model parameters*

LDA-based topic proportion data are provided for three different LDA topic models of the same set of 398,444 publication records. Parameters (reported below) are identical for each of the three models except for the number of topics, which was varied for different runs of the model at 60 topics, 200 topics, and 250 topics. Topic models were generated using a modified version of the ***ldacol*** function from the Matlab Topic Modeling Toolbox 1.3.2^[[2]](#footnote-2)^ (), a Latent Dirichlet Allocation implementation in MATLAB by M. Steyvers and T. Griffiths (2004). ***Ldacol*** uses Gibbs sampling to generate a basic LDA topic model with multi-word phrases (i.e., collocations).

Modifications were made to the Topic Modeling Toolbox to optimize memory usage and for analysis of larger corpora, and the modified version of the toolbox used for this work is available as part of the Text Visualization Toolbox by S. Rebich-Hespanha and J. Hespanha [2].

Topic model parameters were as follows:

• number of topics = [60, 200, 250]

• αT (alpha parameter of the Dirichlet prior on the per-document topic distribution) =

50/number of topics

• αW (alpha parameter of the Dirichlet prior on the per-topic word distribution) =

200/number of unique terms in corpus

• δ (delta parameter of the Dirichlet prior on the per-word next-word-in-collocation

distribution) = 0.1

• γ0 (first gamma parameter of the Beta prior on the per-word probability of next

word being a collocation) = 0.1

• γ1 (second gamma parameter of the Beta prior on the per-word probability of next

word being a collocation) = 0.1

• number of iterations = 1000

The data file is a tab-delimited plain text file that includes the following variables:

*Document characteristics – manifest*

• ‘PY’ = year of publication

• ‘sourceID’ = a numeric categorical identifier that facilitates selection of publications

from individual journals

• ‘synthesis_center_relevant_journal’ = value is 1 if document appeared in one of the

14 journals that is a common publication venue for the research centers of interest

• ‘top_20_disciplinary_journal’ = value is 1 if document appeared in one of the 94 top

disciplinary journals

• ‘top_general_science_journal’ = value is 1 if document appeared in one of the 4 highvisibility general science journals

*Document characteristics – derived*

• ‘NumberAddresses’ = the number of unique addresses identified for the authors

• ‘NumberAuthors’ = the number of authors identified for the publication

• ‘lda60all_nTokens = a measure of document length. For the associated document,

numeric value indicates the number of tokens (i.e., words) from this document that

remained after stopword removal and were used to generate the topic model and

estimate topic proportions. [NOTE: Although 60 is indicated in the variable name,

this value was the same for all topic models, so separate variables were not included

for the 200- and 250-topic models.]

• ‘lda200all_topicWeights_ *NNN*’ = a sequence of 200 fields that contain the topic

proportions associated with each of the topics in the 200-topic model. The numeric

values in the ‘*NNN*’ portion of the variable name indicate the specific topic that

corresponds with the given value, and can be used to cross-reference these

variables with the lists of representative terms for topics provided in the ‘200-

topics-representative-terms.tab’ file. The topic proportion values in all 200 of these

fields sum to 1 for each document. Higher values indicate greater representation of

the associated topic, and zero values indicate absence of the topic.

• ‘lda250all_topicWeights_ *NNN*’ = a sequence of 250 fields that contain the topic

proportions associated with each of the topics in the 250-topic model. The numeric

values in the ‘*NNN*’ portion of the variable name indicate the specific topic that

corresponds with the given value, and can be used to cross-reference these

variables with the lists of representative terms for topics provided in the ‘250-

topics-representative-terms.tab’ file. The topic proportion values in all 250 of these

fields sum to 1 for each document. Higher values indicate greater representation of

the associated topic, and zero values indicate absence of the topic.

• ‘lda60all_topicWeights_*NNN*’ = a sequence of 60 fields that contain the topic

proportions associated with each of the topics in the 60-topic model. The numeric

values in the ‘*NNN*’ portion of the variable name indicate the specific topic that

corresponds with the given value, and can be used to cross-reference these

variables with the lists of representative terms for topics provided in the ‘60-topicsrepresentative-terms.tab’ file. The topic proportion values in all 60 of these fields

sum to 1 for each document. Higher values indicate greater representation of the

associated topic, and zero values indicate absence of the topic.

• ‘maxTopic200all_id’ = numeric categorical variable that indicates the ID number of

the topic with the highest proportion in the 200-topic model for the relevant

document; in other words, the most strongly represented topic for that document in

the 200-topic model.

• ‘maxTopic250all_id’ = numeric categorical variable that indicates the ID number of

the topic with the highest proportion in the 250-topic model for the relevant

document; in other words, the most strongly represented topic for that document in

the 250-topic model.

• ‘maxTopic60all_id’ = numeric categorical variable that indicates the ID number of

the topic with the highest proportion in the 60-topic model for the relevant

document; in other words, the most strongly represented topic for that document in

the 60-topic model.

**stop-words.txt**

This plain text file contains the list of 503 words that were removed from the corpus prior

to analysis. This list was created by building upon the stop word list provided with

MALLET5, a Java-based natural language processing package that includes an

implementation of LDA topic modeling.[3]

**60-topics-representative-terms.txt**

**200-topics-representative-terms.tab**

**250-topics-representative-terms.tab**

These tab-delimited plain text files contain the 40 most representative terms for each topic in each of the three topic models (60 topics, 200 topics, 250 topics). Each text file contains multiple tables. The top table in each file occupies the first three columns and presents a summary of representative terms for all topics in the model.

• ‘topicID’ = contains unique numeric identifiers for topics within the associated topic

model

• ‘weight in docsLDA’ = a measure of how dominant the topic is in the entire corpus;

topics corresponding to large values are more dominant and more frequently

observed.

• ‘representative terms’ = a space-delimited list of the 40 most representative terms

for the associated topic; note that terms that are multi-word collocations are linked

by underscore (_) characters.

Below the top summary table the file contains a sequence of tables, one for each topic (row) in the top table. Each of these tables contains the following columns:

• columns 1 and 3 -- serve as headers for columns immediately to right

• column 2 – refers to ‘topicID’ indicated in rows of top table; header for this column

in column 1

• column 4 – repeats value of ‘weight in docsLDA’ associated with topicID in top table;

header for this column in column 3

• column 5 – weight of term in topic, a measure of how dominant the term is within

the corresponding topic; large values are more dominant and more frequently

observed in documents containing that topic

• column 6 – identifies term to which value in column 5 refers

200 Topic Solution

Top 10+ topics

phylogeny taxa clade species evolution genera genus diversification sequences relationships phylogenetic_relationships clades based lineages phylogenetic_analysis supported characters molecular_phylogeny phylogenies monophyletic inferred monophyly systematics analyses biogeography group morphology support resolved origin trees speciation radiation taxonomy divergence taxon combined classification phylogenetic_analyses including

populations genetic_diversity loci population markers genetic_variation microsatellites individuals genetic_structure population_structure microsatellite_loci differentiation genetic_differentiation microsatellite_markers inbreeding_depression inbreeding alleles heterozygosity f genetic_variability polymorphism diversity genotypes population_genetics polymorphic detected microsatellite expected observed marker gene_flow isolated isolation genetic_drift aflp samples eight sampled allele_frequencies allozymes

structure protein binding domain residues crystal_structure complex structures proteins folding bound interactions conformation peptide interaction mechanism residue complexes ligand domains bind recognition loop dimer interface helix binds form ligands subunit reveals binding_site affinity solution fold assembly peptides stability conformations molecular_dynamics

males females male female sexual_selection sex sex_ratio sexes mate_choice mating evolution sexual_dimorphism males_females male_female paternity reproductive_success mating_system mates offspring sex_determination sex_ratios sperm_competition sexual_reproduction female_choice sex_specific courtship selection sexual_conflict sex_allocation mating_success mate sex_differences sperm mating_systems polyandry sex_chromosomes hypothesis gender copulation dimorphism

trees stands tree stand growth thinning height forest tree_species regeneration forests diameter plots pine stems douglas_fir branches crown basal_area spruce mature oak beech biomass foliage plantations stem tree_growth needles branch norway_spruce scots_pine volume conifers plot stand_structure density conifer dbh needle

database alignment tool sequences tools based information data software search program sequence developed algorithm databases annotation automated visualization prediction alignments system analysis set programs algorithms application datasets users web existing reads bioinformatics generation platform sequencing automatic blast user query mapping

activation phosphorylation pathway signaling activity kinase protein regulation inhibition degradation dependent mediated activated phosphorylated induced cells ras receptor akt regulates regulated camp kinases signaling_pathway signal_transduction ubiquitination downstream stat3 erk signaling_pathways show pathways inhibitor proteasome pka activates src mtor inhibits expression

task memory learning brain processing performance stimuli subjects perception attention related language stimulus fmri participants activity recognition tasks activation information faces reward face training cortex prefrontal_cortex regions object words left hand objects action amygdala representation emotional working_memory experience cognition perceptual

expression cells cancer tumors p53 breast_cancer metastasis tumor cancer_cells progression normal human tumor_cells melanoma proliferation tumorigenesis tumor_suppressor cancers overexpression apoptosis cell_lines myc tumor_growth prostate_cancer carcinoma vivo egfr induced hcc glioma tumour pten oncogene pathway metastatic regulation carcinogenesis rb tumor_progression inhibition

populations phylogeography mtdna species haplotypes mitochondrial_dna divergence lineages speciation subspecies genetic_diversity pleistocene history patterns gene_flow refugia sequences population cryptic complex differentiation genetic_structure lineage genetic_variation population_structure range diversity region diversification mitochondrial individuals haplotype historical suggest inferred colonization expansion distribution revealed glacial_refugia

formation teeth fossil evolution fossils modern extant fossil_record specimens taxa preserved morphology skull features specimen extinct early tooth bones derived evidence ma new record characters hominin basin living earliest modern_humans primitive hominid lower skeleton origin cave including dinosaurs material dental

diet diets fish fed growth protein levels feed dietary feeding meal level fatty_acids supplementation supplemented digestibility formulated ratio content lipid rainbow_trout fatty_acid_composition lipids fish_meal growth_performance weight feed_intake nutrition effect feeds requirement groups fm replacement inclusion soybean_meal higher weight_gain body_composition fish_fed

science research knowledge management policy project implementation scientists program people information issues process monitoring programs development projects students challenges participation attitudes education practice conflict institutions forestry stakeholders public practices planning governance traditional problems sustainability support perceptions goals scientific political communication

transcription promoter methylation complex chromatin promoters transcription_factor dna expression transcription_factors gene repression binding silencing gene_expression regulation genes dna_methylation specific acetylation vivo activity show heterochromatin yeast required dependent complexes function saccharomyces_cerevisiae repressor activation enhancer protein domain binding_sites transcriptional_regulation methylated regulated transcriptional_activation

species diversity communities species_richness community biodiversity richness abundance patterns assemblages community_structure species_composition productivity composition taxa species_diversity disturbance community_composition assemblage gradient evenness similarity species_specific diverse gradients sampled relationships structure nestedness ecology community_assembly community_level species_rich abundances community_ecology dominance relationship complementarity dominant guild

enzyme enzymes substrate activity reaction biosynthesis synthesis substrates reactions oxidation mechanism pathway escherichia_coli formation complex product cleavage heme active_site active catalysis reduction purification catalyzed synthase hydrolysis products protease complexes acid purified intermediate enzyme_activity cofactor catalytic reductase protein proteases step conversion

genes expression gene gene_expression identified transcripts gene_flow expressed regulated transcriptome analysis regulation genome identification genes_involved tissues microarray putative transcript involved differentially_expressed pathways specific pathway cdna genes_encoding microarrays identify gene_family encoding candidate transcription related differential including gene_regulation set mrna microarray_data gene_expression_profiles

states state spectroscopy phase atoms electrons spin dynamics coherent transition coupling molecules measurements electronic optical systems single spectra scattering system lattice excitation charge superconducting light superconductivity properties report relaxation atom field polarization band laser observed observation demonstrate quantum transport materials

Examples of topics removed

number total values ratio average volume proportion calculated estimated measured numbers determined ratios relative percentage minimum amount absolute cumulative highest obtained fraction units measure unit lowest estimate volumes proportional actual proportions equal total_number counting terms quantity calculate varied quantified sum

three four five six one seven eight nine two ten three_species remaining fourth identified three_dimensional found species twelve eleven representing examined selected tested four_species ea analyzed three_groups thirteen showed study fifteen included three_types studied three_times fourteen represented three_distinct three_sites sixteen

time long_term duration long short_term short longer time_series times shorter long_lived temporal long_distance interval temporal_variation intervals long_range prolonged short_lived periods repeated extended lag time_scales durations temporal_variability time_scale long_lasting longest temporal_changes long_distance_dispersal long_term_effects long_term_potentiation time_points temporal_patterns time_spent time_period time_dependent persistent long_periods

three four five six one seven eight nine two ten three_species remaining fourth identified three_dimensional found species twelve eleven representing examined selected tested four_species ea analyzed

1 2 3 1_2 1_percent 1_3 2_3 4 1_year 2_percent 1_4 ho 2_years 2_4 1_1 pla 1_5 pu 1_expression 1_6 2_weeks sn 2_5 3_4 1_h levels 1_week cav 1_7 egr found pim cells observed 1_000 species 1_day 2_months 1_8 identified

3 4 5 2 1 6 7 8 9 5_percent 3_4 11 10 1_2 5_ht 3_percent 4_percent 4_5 5_years 5_6 3_years 6_percent 12 6_7 3_5 2_3 6_months 7_percent 7_8 4_years 8_percent 4_6 8_9 1_3 5_7 4_weeks 2_percent 3_utr 3_months 2_years

14 12 11 13 15 10 16 19 17 18 21 23 22 20 27 50_percent 26 29 25 24 31 32 28 33 41 34 30 37 38 20_percent 42 36 35 39 10_percent 44 25_percent 43 40 average

_______________________________________

[1] 1 D. Blei, A. Ng, and M. Jordan (2003). Latent Dirichlet allocation. *Journal of Machine Learning Research*, 3:993-1022

[2] Rebich-Hespanha, S. & Hespanha, J. *Text Visualization Toolbox — a MATLAB toolbox to visualize large corpus of documents.*  http://web.ece.ucsb.edu/~hespanha/software/textvistools.html, Feb.

2010.

[3] McCallum, Andrew Kachites. *MALLET: A Machine Learning for Language Toolkit*.

http://mallet.cs.umass.edu. 2002.

**Author contributions**

S. Rebich-Hespanha helped to conceive the study, coordinated journal selection and data

collection, performed the analyses, and documented the work.

S. E. Hampton helped to conceive the study and provided feedback during the journal

selection process.

B. Adams downloaded the journal publication data.

J. P. Hespanha assisted with data preparation and analysis.

E. J. Hackett and J. N. Parker conceived the study and led the working group discussion.

1. http://www.eigenfactor.org/ [↑](#footnote-ref-1)
2. http://psiexp.ss.uci.edu/research/programs_data/toolbox.htm [↑](#footnote-ref-2)
